# Specificity for the correlation between the body surface and viscera in the pathological state of COPD: A prospective, controlled, and assessor-blinded trial

**DOI:** 10.3389/fphys.2023.1051190

**Published:** 2023-04-19

**Authors:** Yongliang Jiang, Hantong Hu, Xiaofen He, Xiaoyu Li, Yajun Zhang, Jiali Lou, Yuanyuan Wu, Junfan Fang, Xiaomei Shao, Jianqiao Fang

**Affiliations:** ^1^ Key Laboratory of Acupuncture and Neurology of Zhejiang Province, Department of Neurobiology and Acupuncture Research, The Third Clinical Medical College, Zhejiang Chinese Medical University, Hangzhou, China; ^2^ Department of Acupuncture and Moxibustion, The Third Affiliated Hospital of Zhejiang Chinese Medical University, Hangzhou, Zhejiang, China

**Keywords:** site specificity, infrared thermography, comparative study, acupuncture, skin physiology

## Abstract

**Background:** The association between the body surface and viscera remains obscure, but a better understanding of the body surface-viscera correlation will maximize its diagnostic and therapeutic values in clinical practice. Therefore, this study aimed to investigate the specificity of body surface-viscera correlation in the pathological state.

**Methods:** The study subjects included 40 participants with chronic obstructive pulmonary disease (COPD) in the COPD group and 40 age-matched healthy participants in the healthy control group. Laser Doppler flowmetry, infrared thermography, and functional near-infrared spectroscopy were respectively adopted to measure 1) the perfusion unit (PU), 2) temperature, and 3) regional oxygen saturation (rSO_2_) of four specific sites distributed in the heart and lung meridians. These three outcome measures reflected the microcirculatory, thermal, and metabolic characteristics, respectively.

**Results:** Regarding the microcirculatory and thermal characteristics of the body surface, the PU and temperature of specific sites on the body surface [i.e., Taiyuan (LU9) and Chize (LU5) in the lung meridian] in the COPD group were significantly increased compared with healthy controls (*p* < 0.05), whereas PU and temperature of other sites in the heart meridian [i.e., Shenmen (HT7) and Shaohai (HT3)] did not change significantly (*p >* 0.05). Regarding the metabolic characteristics, rSO_2_ of specific sites in the lung meridian [i.e., Taiyuan (LU9) and Chize (LU5)] and Shaohai (HT3) of the heart meridian in the COPD group was significantly decreased compared with healthy controls (*p* < 0.01), whereas rSO_2_ of Shenmen (HT7) in the heart meridian did not change significantly (*p >* 0.05).

**Conclusion:** In the disease state of COPD, the microcirculatory, thermal, and metabolic characteristics of specific sites on the body surface in the lung meridian generally manifest more significant changes than those in the heart meridian, thereby supporting relative specificity for the body surface-viscera correlation in the pathological state.

## 1 Introduction

According to theories of modern medicine, pathological states of visceral organs can manifest changes in specific sites on the body surface, such as referred somatic hypersensitivity (Han and Lee, 2009). Especially for visceral pain, it tends to induce referred pain located in specific regions of the body surface. A common example is that during a heart attack, pain is typically felt in the arm, neck, and back rather than in the chest and heart. Such body surface-viscera correlations can generally be explained by the segmental innervation theory. In detail, visceral disorders can evoke central sensitization with hypersensitivity and expansion in the number and size of receptive fields, being segmentally predominant at the level of the affected viscera, and produce muscle contraction, sympathetic activation, and antidromic activation of afferent fibers by visceral-somatic reflex, which induces neurogenic inflammation (Giamberardino, 2003). Referred pains are generally manifested on the same segments of the spinal cord that visceral afferents project to (Bogduk, 2009).

Nevertheless, the segmental innervation theory cannot fully explain all the phenomena reflecting the body surface-viscera correlation. The rules of the body surface-viscera correlation are very complex and its underlying mechanisms are still not completely understood (Luz et al., 2015; Gao et al., 2021). A better understanding of the body surface-viscera correlation will maximize its diagnostic and therapeutic values. For instance, abnormal body temperature in specific sites assessed by modern techniques is a useful indicator of visceral diseases (Lahiri et al., 2012) and the early detection of it can aid in the diagnosis of diverse diseases, such as tumors (Mambou et al., 2018) and liver diseases (Ramírez-Elías et al., 2017). In addition, stimulating peripheral regions on the body surface can regulate pathological states of visceral organs, and a typical example is the emerging program entitled “Stimulating Peripheral Activity to Relieve Conditions (SPARC)” (Waltz, 2016) funded by The National Institutes of Health (NIH) in recent years. The SPARC program attempts to accelerate the development of therapeutic devices that modulate electrical activity on the body surface to improve organ function (Waltz, 2016). Given the significance of the body surface-viscera correlation, some researchers have put forward the concept of “body surface medicine,” which emphasizes the diagnostic and therapeutic values of the body surface.

Similarly, in traditional Chinese medicine (TCM), according to its classic theory called “meridian-viscera correlation,” pathological changes of viscera can manifest in specific sites on the body surface distributed in relevant meridians, especially acupoints (Zhao, 2018); in turn, stimulating these involved sites on the body surface can regulate pathological states of viscera (Liu et al., 2018). For example, cardiovascular diseases can manifest in the heart meridian and be treated with acupoints distributed on the heart meridian (Shi et al., 2018). Moreover, it is likely to have relative specificity for the association between viscera and meridians. For instance, given that the heart meridian connects the heart directly and the lung meridian connects the heart indirectly according to TCM, when treating cardiovascular diseases, the therapeutic effect of stimulating sites on the body surface located in the heart meridian may be superior to sites located in the lung meridian (Zhao et al., 2019). Despite numerous previous studies (Kong et al., 2009; Rong et al., 2011; Fang et al., 2017), the law of meridian-viscera correlation is very complicated and its exact underlying mechanisms remain inconclusive. Does one viscus correspond to one meridian? Or does one viscus correspond to multiple meridians? Or do multiple viscera correspond to one meridian? Answers to such questions remain obscure (Zhu, 2015). In this scenario, further in-depth researches are urgently needed. In addition, results of previous studies in this field often contain subjective elements and most of them only rely on a single technique.

Taken together, this study selected acupoints in the lung meridian as the research entry, because acupoints tend to be more sensitive sites on the body surface to manifest pathological changes of viscera than other surface sites. By detecting diverse biological characteristics of the body surface using three modern techniques simultaneously, this study aimed to investigate the specificity of the body surface-viscera correlation in the pathological state.

## 2 Materials and methods

### 2.1 Study designs

This was a prospective clinical controlled trial. To investigate the specificity of body surface-viscera correlation as far as possible, three kinds of biological characteristics of specific sites on the body surface were detected by corresponding modern techniques, namely, the microcirculatory characteristics measured by laser doppler flowmetry (LDF), thermal characteristics of measured by infrared thermography (IRT), and metabolic characteristics measured by functional near-infrared spectroscopy (fNIRS).

### 2.2 Ethical approval and trial registration

Ethical approval (approval No: ZSLL-KY-2019-001-01) was obtained from the Ethics Committee of the Third Affiliated Hospital of Zhejiang Chinese Medical University. All participants were fully informed of this trial and informed consent was signed. In addition, we registered the protocol of this trial in the Clinicaltrials registry (registration number NCT05690217).

### 2.3 Study subjects

The study subjects consisted of COPD patients and healthy control participants. To exclude the age-related influence on biological characteristics of the body surface between two groups according to our previous study (Zhang et al., 2022), the healthy controlled participants were age-matched with the COPD patients. By referring to similar studies (Dams-O’Connor et al., 2013; Xie et al., 2013), the details of the operation of including “age-matched” controls were described as follows. When a COPD patient was included in the trial, we would synchronously recruit and enroll an age-matched (±3 years) healthy subject from the medical examination center in our hospital or the local communities via advertisements.

#### 2.3.1 Diagnostic criteria of COPD

The diagnostic criteria of COPD were based on criteria proposed by “the Global Initiative for Chronic Obstructive Lung Disease (GOLD)” in 2017 (Vogelmeier et al., 2017), in which persistent airflow obstruction is confirmed according to the pulmonary function test (i.e., post-bronchodilator FEV1/FVC<0.70).

#### 2.3.2 Inclusion criteria

COPD participants were eligible if they 1) satisfied the diagnostic criteria of COPD; 2) were in the stable phase of COPD; 3) were ≤20 age ≤75 years. Healthy participants were eligible if they 1) were healthy subjects who provided a medical examination report within six preceding months for confirming their health conditions; 2) were age-matched with the COPD group. Besides, an additional criterion for both COPD participants and healthy subjects was that they should fully understand the study protocol and sign written informed consent.

#### 2.3.3 Exclusion criteria

COPD patients were excluded if they 1) had other major lung diseases, such as bronchiectasis, pneumothorax and lung tumors; 2) had concomitant heart diseases; 3) had serious concomitant diseases in major systems that were not controlled effectively; 4) had mental diseases, a history of drug abuse and alcohol dependence; 5) were lactating or pregnant. Healthy subjects were excluded if they 1) had mental illness, a history of drug abuse and alcohol dependence; 2) were lactating or pregnant. In addition, another shared exclusion criterion for both COPD participants and healthy subjects was that they had scarring, hyperpigmentation, redness, swelling and heat in the skin around the measurement sites, which would affect the accuracy of examinations.

### 2.4 Sample size calculations

This study was a trial aiming to measure diverse biological characteristics in COPD patients and healthy subjects. Compared with common clinical trials that intend to evaluate the effectiveness of a specific therapy, there is no consensus on the standardization of sample size estimation for such studies. Thus, the sample size estimation was mainly according to similar trials (Hsiu et al., 2007; Raith et al., 2013; Fu et al., 2016) and actual research conditions of our study during different study phases. Finally, the total sample size was 80, with 40 subjects in the COPD group and 40 subjects in the healthy control group.

### 2.5 Grouping

Followed by screening based on their corresponding inclusion and exclusion criteria, two kinds of eligible subjects were assigned to the COPD group and the healthy control group, respectively.

### 2.6 Blinding

Participants were not blinded in this study. Examinations of biological characteristics and data collection were performed by specialized personnel who were not aware of the grouping. Statistical analysis was also performed by third-party statisticians who were unaware of the grouping.

### 2.7 Procedure for examinations of biological characteristic

Specifically, the microcirculatory characteristic of the body surface was assessed by LDF. The thermal characteristic of the body surface was assessed by IRT. And the metabolic characteristic of the body surface was assessed by fNIRS.

#### 2.7.1 Examination conditions

With references to similar studies (Hsiu et al., 2007; Wang et al., 2013; Jo and Kim, 2016) in this field and our previously published study (Lou et al., 2020), the establishment of examination conditions was described in detail as follows. One week before examinations, all subjects refrained from medications that could affect microcirculation and vasoreactivity for 1 week. One day before examinations, all subjects could not consume stimulating drinks (e.g., tea, alcohol, and coffee) and smoke. Besides, exercise and diet were forbidden within 1 h before examinations. Testing time was fixed at 9:00 a.m.–11:00 a.m. Participants were asked to stabilize for 30 min in a supine position in the experimental room. Before examinations of biological characteristics, fundamental physiological parameters, including body temperature, heart rate, blood pressure, and respiratory rate, were also measured. Throughout the full measurement periods, all subjects were informed to maintain normal breath and avoid limb movement. In addition, for female subjects, examinations should be performed outside the menstrual cycle to avoid interference induced by basal body temperature.

#### 2.7.2 Examination environment

An experimental room was set up for examinations. The temperature for the examination environment was strictly controlled at 26°C and the humidity was controlled between 50% and 60% (Lou et al., 2020).

#### 2.7.3 Measurement sites of the body surface

**FIGURE 1:**
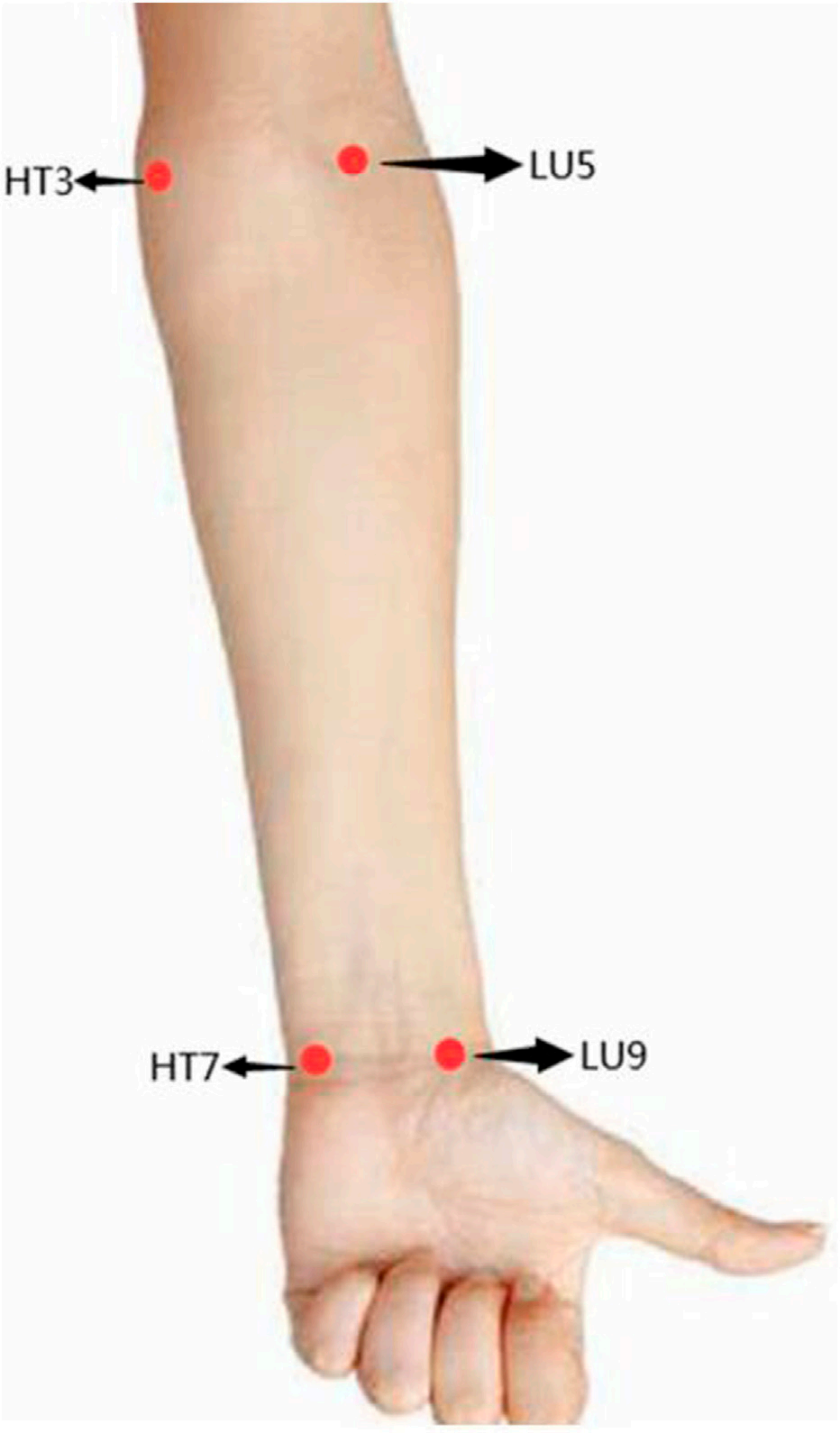
The schematic diagram for four measurement sites on the body surface.

**FIGURE 2:**
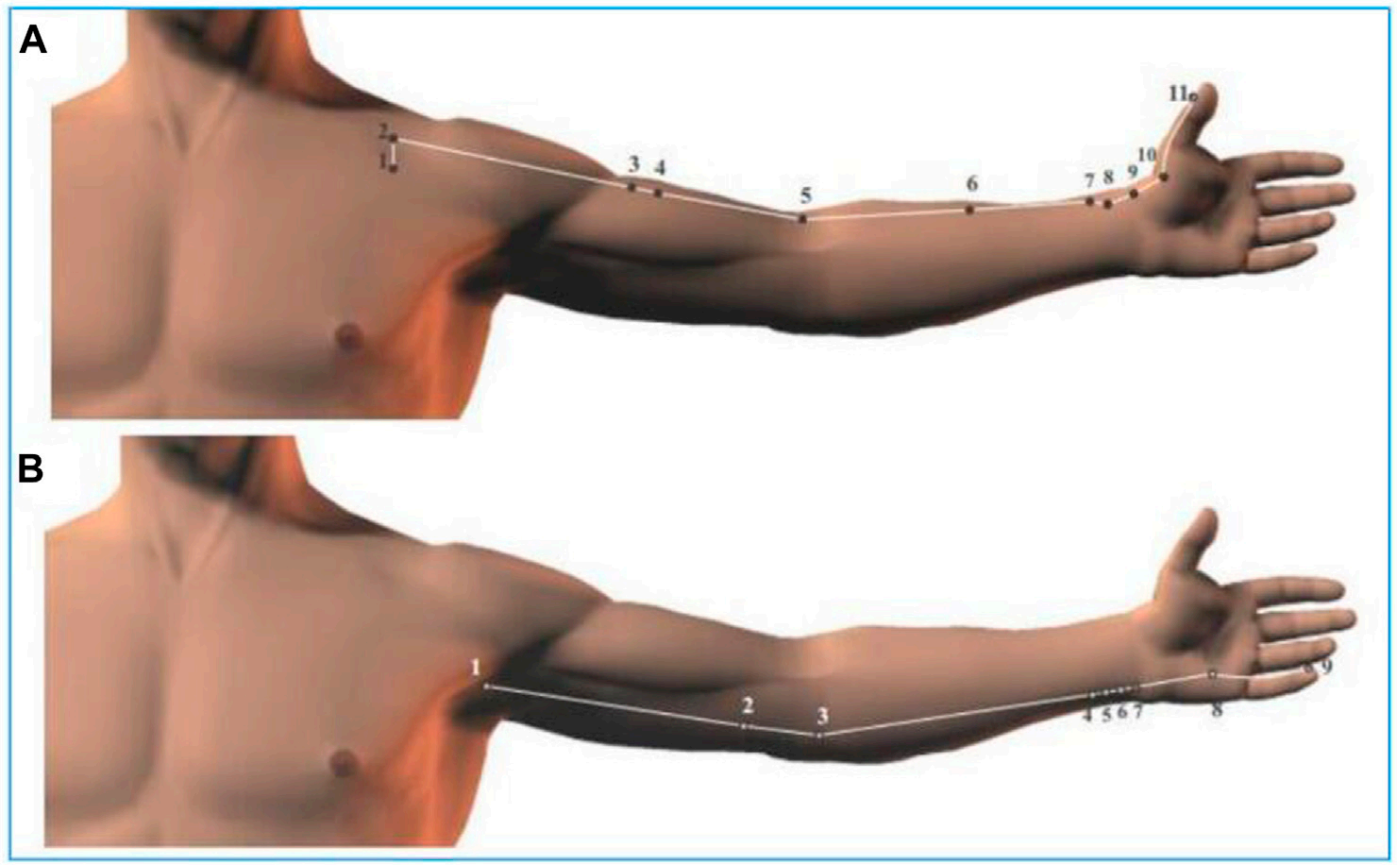
The distribution path of the heart and lung meridian on the body surface. **(A)** The lung meridian; **(B)** the heart meridian. The Arabic number represents the number of the corresponding acupoint on the meridian in sequence.

As presented in Figure 1, the measurement sites of the body surface were four specific sites located in the heart and lung meridians, including Shenmen (HT7), Shaohai (HT3), Taiyuan (LU9), and Chize (LU5). The location and regional anatomy of the four measurement sites were described in detail in Table 1. In addition, the running routes of the lung meridian and the heart meridians on the body surface were shown in Figures 2A, B, respectively.

**TABLE 1:** Location and regional anatomy of four measurement sites.

Measurement sites	Location	Regional anatomy and the innervation
Shenmen (HT7)	On the anteromedial aspect of the wrist, radial to the flexor carpi ulnaris tendon, on the palmar wrist crease	Major anatomical layers include 1) skin and subcutaneous tissue; 2) ulnar flexor carpi tendons; 3) ulnar nerve trunk and ulnar artery/ulnar vein
Shaohai (HT3)	On the anteromedial aspect of the elbow, just anterior to the medial epicondyle of the humerus, at the same level as the cubital crease	Major anatomical layers include 1) skin and subcutaneous tissue; 2) pronator teres muscle; 3) the median nerve trunk, ulnar artery and ulnar vein; 4) brachialis muscle
Taiyuan (LU9)	On the anterolateral aspect of the wrist, between the radial styloid process and the scaphoid bone, in the depression ulnar to the abductor pollicis longus tendon	Major anatomical layers include 1) skin and subcutaneous tissue; 2) between the radial flexor carpi tendons and extensor pollicis longus tendon; 3) radial artery and radial vein
Chize (LU5)	On the anterior aspect of the elbow, at the cubital crease, in the depression lateral to the biceps brachii tendon	Major anatomical layers include 1) skin and subcutaneous tissue; 2) brachioradialis muscle; 3) radial nerve trunk, anterior branches of radial collateral artery and vein

#### 2.7.4 Procedures

All included participants would receive LDF, IRT, and fNIRS examinations in sequence. To avoid the potential interexperiment interference, a 30-min interval period was implemented between different examinations.

(1) Procedures for the LDF examination

**FIGURE 3:**
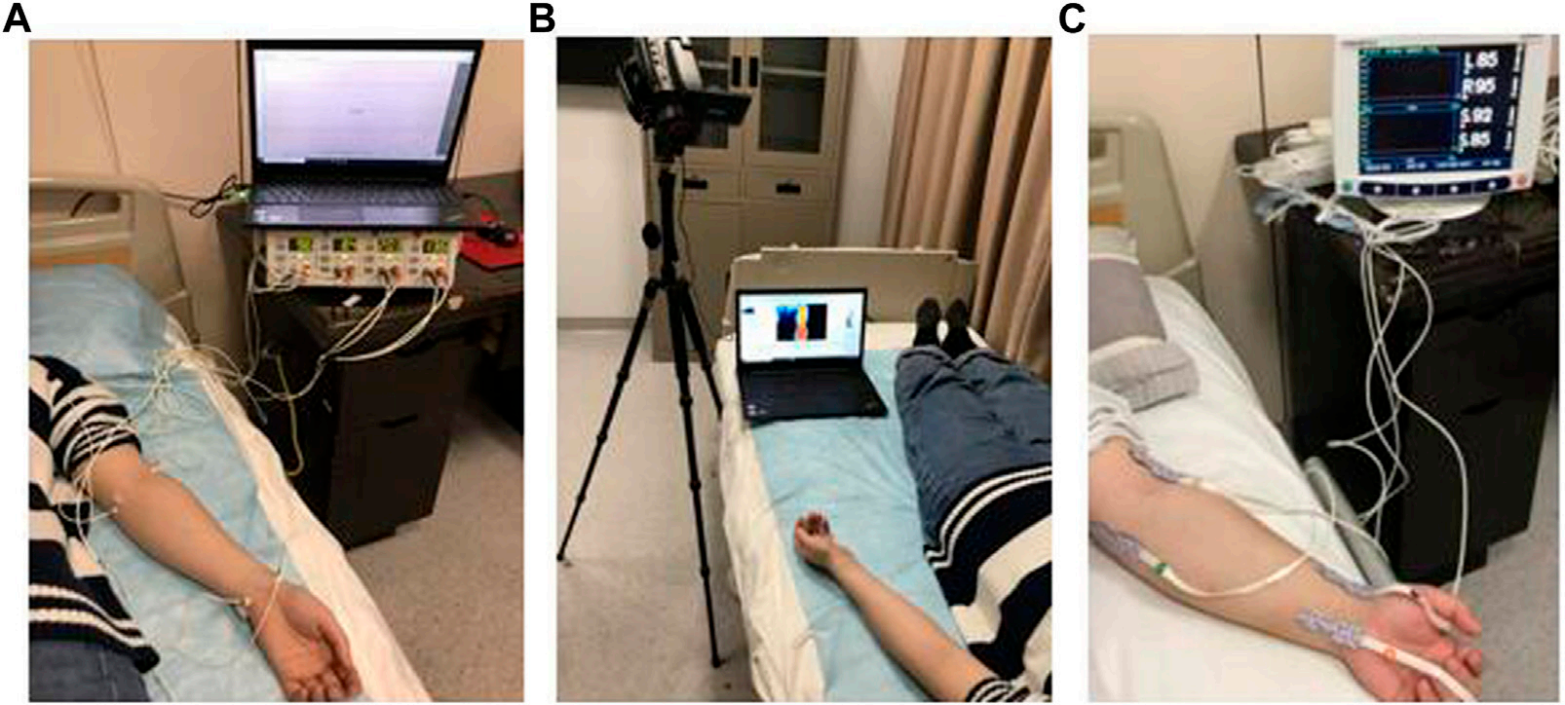
The schematic of the experiment. **(A)** LDF detection; **(B)** IRT detection; **(C)** fNIRS detection.

The schematic of the LDF examination is shown in Figure 3A. In detail, a four-channel LDF (PeriFlux System 5000, Sweden) was used to measure the microcirculatory flux of the body surface, which was quantified as the parameter entitled blood perfusion units (PU) using Perisoft for Windows. The probes (model: Probe 407) were left at four aforementioned measurement sites. PU was constantly recorded for 10 min.

(2) Procedures for the IRT examination

The schematic of the IRT examination is shown in Figure 3B. In detail, a thermograph (NEC InfRec R450, Avio Infrared Technologies Co., Ltd., Tokyo) was used to take infrared thermal images of the body surface. IRT examination lasted for 10 mins, with one thermal image taken per minute. All infrared thermal images were analyzed by the software InfRec Analyzer NS9500 (Avio Infrared Technologies Co., Ltd., Tokyo). For each image, the temperature of the corresponding sites was measured and then averaged.

(3) Procedures for the fNIRS examination

The schematic of the fNIRS examination is shown in Figure 3C. In detail, a four-channel Oximeter (INVOS 5100C, Somanetics CorpUnited States., Troy, United States) was used to detect the metabolic characteristic of the body surface. The probes were placed at four aforementioned measurement sites synchronously to monitor oxygen saturation for 10 mins, which was quantified as the parameter entitled regional oxygen saturation (rSO_2_) using the INVOS Analytics tool.

### 2.8 Outcome measures

(1) The outcome of the microcirculatory characteristics of specific sites was PU.
(2) The outcome of the thermal characteristics of specific sites included infrared thermal images, mean acupoint temperature, and mean meridian temperature.
(3) The outcome of the metabolic characteristics of specific sites was rSO_2_.

### 2.9 Statistical analysis

Statistical analysis was performed using SPSS 25.0 for Windows (SPSS Inc., Chicago, IL, United States). All data were first tested for normality. If normality was met, continuous variables were described as the mean and standard error of the mean (mean ± SEM). The independent sample *t*-test was used for between-group comparisons, and the paired *t*-test was used for within-group comparisons. If normality was not met, continuous variables were expressed by median (quartiles) [M (Q1, Q3)]. The Mann-Whitney U rank sum test was used for between-group comparisons and the Wilcoxon signed rank test was used for within-group comparisons. A *p*-value of less than 0.05 will be considered statistically significant.

## 3 Results

### 3.1 Baseline characteristics of included subjects

Forty subjects (29 males and 11 females, mean age was 65.18 ± 6.71 years) were included in the COPD group. Forty healthy subjects (20 males and 20 females, mean age was 62.98 ± 5.27 years) were included in the healthy control group. There was no significant difference in the mean age between the two groups (*p* > 0.05).

### 3.2 Comparison of microcirculatory characteristics between two groups

**FIGURE 4:**
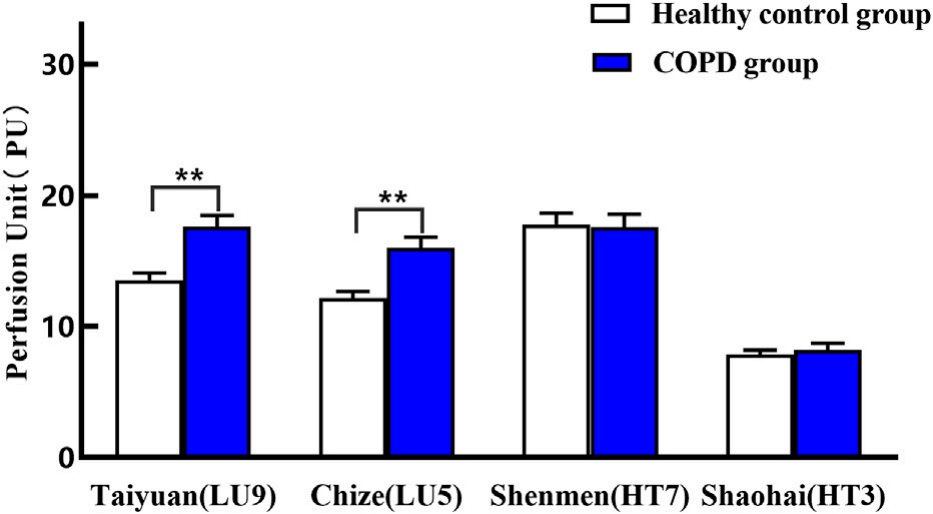
Comparison of PU on specific acupoints of heart and lung meridians between two groups. **p < 0.01 compared with the healthy control group.

As shown in Table 2 and Figure 4, compared with the healthy control group, PU of Taiyuan (LU9)/Chize (LU5) of the lung meridian in the COPD group increased significantly (*p* < 0.01). The greatest increase was observed in Taiyuan (LU9) with a mean increase of 4.10 units, which is the Yuan-Primary point of the lung meridian. Yet, the difference in PU in Shenmen (HT7)/Shaohai (HT3) between the two groups was not statistically significant (*p* > 0.05).

**TABLE 2:** Comparison of PU on specific acupoints of heart and lung meridians between the two groups (mean ± SEM).

Group	Lung meridian		Heart meridian	
	Taiyuan (LU9)	Chize (LU5)	Shenmen (HT7)	Shaohai (HT3)
Healthy control group	13.51 ± 0.57	12.17 ± 0.50	17.77 ± 0.87	7.86 ± 0.32
COPD group	17.61 ± 0.87**	16.00 ± 0.82**	17.56 ± 1.00	8.19 ± 0.51

**p < 0.01 compared with the healthy control group.

### 3.3 Comparison of thermal characteristics between two groups

(1) Comparison of infrared thermal images

**FIGURE 5:**
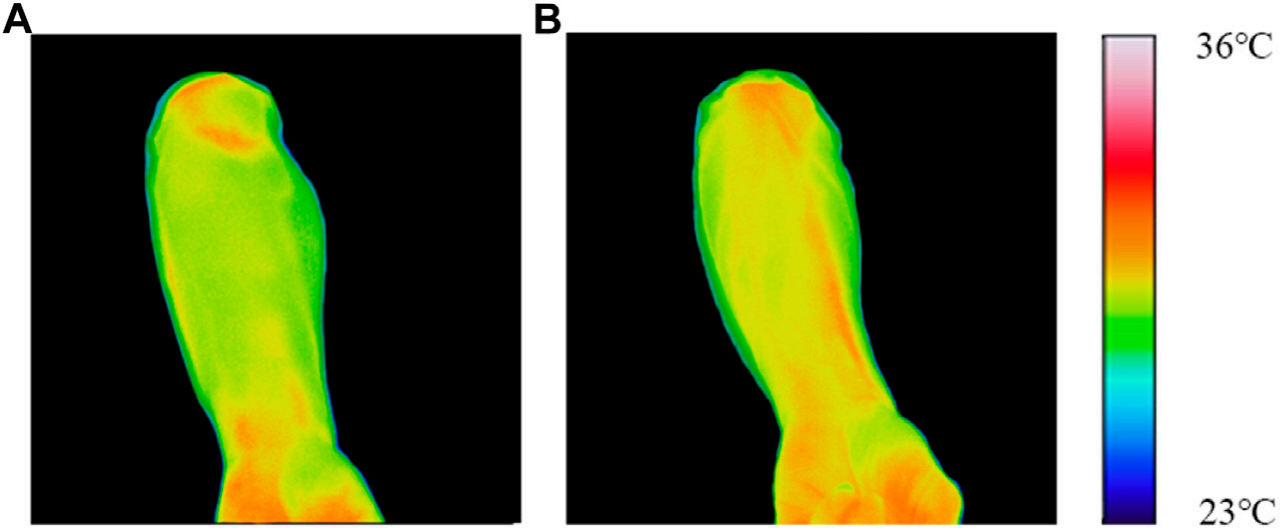
Typical infrared thermal images of typical subjects. **(A)** Healthy control group; **(B)** COPD group.

As shown in Figure 5A, the infrared thermal images of the forearm in the healthy control group showed moderate temperatures in the circulation regions of the heart and lung meridians. There were no abnormal high-temperature points or strip-like high-temperature areas in the circulation regions of both meridians. Some physiological high-temperature areas (shown in red color) were mainly distributed in the wrist and elbow joints, which may be related to some large blood vessels or muscle bundles traveling in these areas.

As shown in Figure 5B, compared with healthy controls, significant hyperthermia areas (shown in red color) were observed in the circulation regions of the lung meridian (located in the radial side of the medial forearm) among some COPD patients, representing red dots, sheets or strips; while no abnormal hyperthermia areas were found in the circulation regions of the heart meridian circulation (located in the ulnar side of the medial forearm).

(2) Comparison of mean acupoint temperature

**FIGURE 6:**
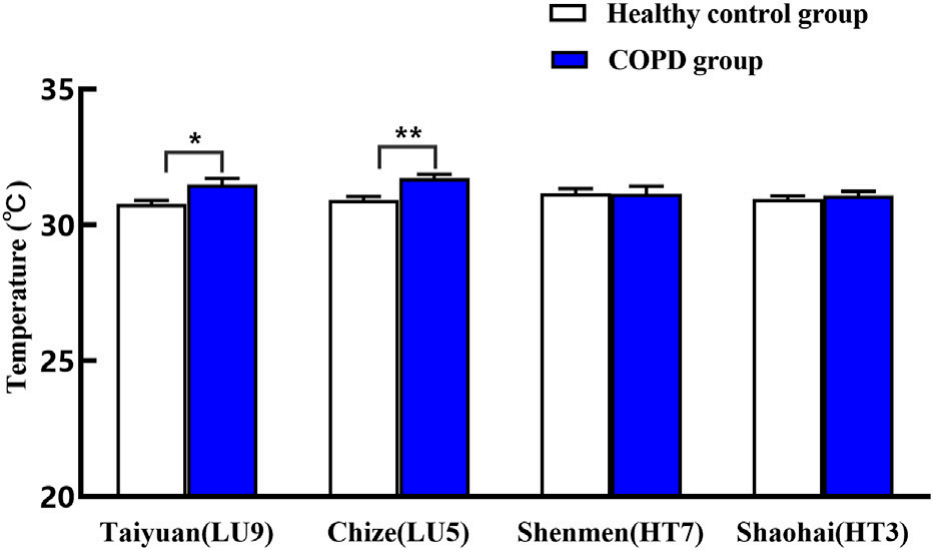
Comparison of mean temperature on specific sites of heart and lung meridians between two groups. **p < 0.01 and *p < 0.05 compared with the healthy control group.

As shown in Table 3 and Figure 6, compared with the healthy control group, the temperature of Taiyuan (LU9)/Chize (LU5) of the lung meridian in the COPD group increased significantly (*p* < 0.01, *p* < 0.05), with a mean increase of 0.70 (°C) in Taiyuan and 0.79 (°C) in Chize, respectively. Yet, the difference in temperature in Shenmen (HT7)/Shaohai (HT3) of the heart meridian between the two groups was not statistically significant (*p* > 0.05).

(3) Comparison of mean meridian temperature

**TABLE 3:** Comparison of mean temperature on specific acupoints of heart and lung meridians between the two groups (mean ± SEM).

Group	Lung meridian		Heart meridian	
	Taiyuan (LU9)	Chize (LU5)	Shenmen (HT7)	Shaohai (HT3)
Healthy control group	30.78 ± 0.14	30.93 ± 0.12	31.18 ± 0.16	30.96 ± 0.11
COPD group	31.48 ± 0.24*	31.72 ± 0.15**	31.15 ± 0.28	31.08 ± 0.16

**p < 0.01 and *p < 0.05 compared with the healthy control group.

As shown in Table 4, there was no statistically significant difference in the mean temperature of the heart and lung meridians in the forearm between the two groups (*p* > 0.05).

**TABLE 4:** Comparison of mean meridian temperature in the forearm between two groups (mean ± SEM).

Group	Lung meridian	Heart meridian
Healthy control group	31.00 ± 0.14	31.12 ± 0.13
COPD group	30.58 ± 0.22	30.98 ± 0.20

### 3.4 Comparison of metabolic characteristics between two groups

**FIGURE 7:**
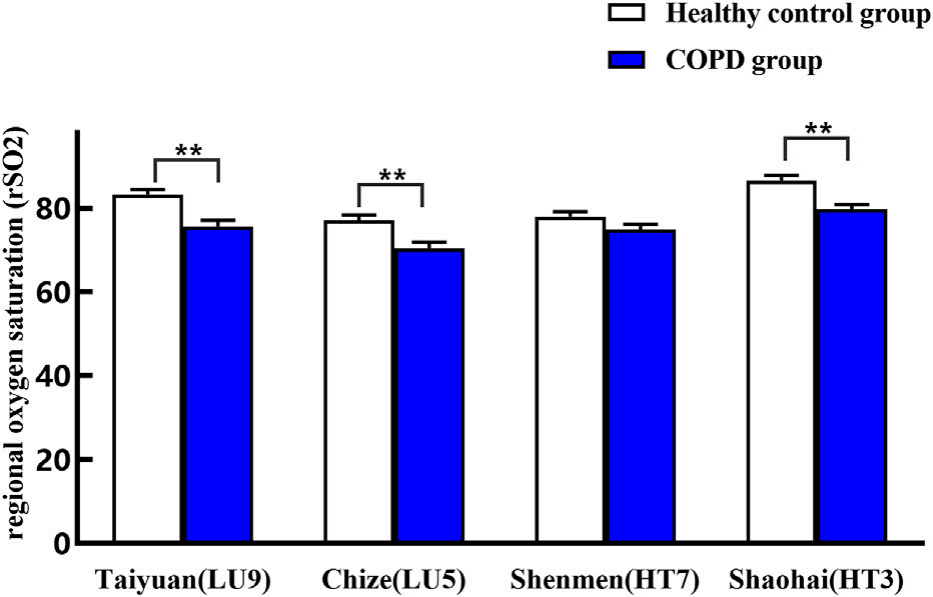
Comparison of rSO_2_ on specific acupoints of heart and lung meridians between the two groups. **p < 0.01 compared with the healthy control group.

As shown in Table 5 and Figure 7, rSO_2_ of four specific acupoints of the lung and heart meridians in the COPD group showed a decreasing trend. Compared with the healthy control group, rSO_2_ of Taiyuan (LU9)/Chize (LU5) of the lung meridian and Shaohai (HT3) of the heart meridian in the COPD group decreased significantly (*p* < 0.05), with a mean decrease of 7.63 (%) in Taiyuan (LU9), 6.69 (%) in Chize (LU5), and 6.76 (%) in Shaohai (HT3), respectively. Thus, the greatest decrease in rSO_2_ was observed in Taiyuan (LU9), which is the Yuan-Primary point of the lung meridian. Yet, the difference of rSO_2_ in Shenmen (HT7) of the heart meridian between the two groups, which is the Yuan-Primary point of the heart meridian, was not statistically significant (*p* > 0.05).

**TABLE 5:** Comparison of rSO_2_ on specific acupoints of heart and lung meridians between the two groups (mean ± SEM).

Group	Lung meridian		Heart meridian	
	Taiyuan (LU9)	Chize (LU5)	Shenmen (HT7)	Shaohai (HT3)
Healthy control group	83.29 ± 1.14	77.16 ± 1.26	78.06 ± 1.14	86.58 ± 1.26
COPD group	75.66 ± 1.51**	70.47 ± 1.51**	74.96 ± 1.25	79.82 ± 1.01**

**p < 0.01 compared with the healthy control group.

## 4 Discussion

### 4.1 Analysis of study results and comparison with similar studies

In terms of the microcirculatory and thermal characteristics of the body surface during the pathological state of the lung, we found that PU and temperature of specific sites on the body surface in the lung meridian [i.e., Taiyuan (LU9)/Chize (LU5)] were significantly increased compared with healthy controls, whereas PU and temperature of corresponding sites in the heart meridian [i.e., Shenmen (HT7)/Shaohai (HT3)] did not change significantly, suggesting that blood perfusion and temperature tended to increase in specific sites on the body surface of meridians that are directly related to the viscus in pathological states. The current results in this respect are similar to previous trials. For example, one clinical trial (Yang et al., 2020) compared the microcirculatory characteristics of acupoints in patients of chronic gastritis with healthy control subjects, and it revealed that patients with chronic gastritis had increased PU at specific acupoints or sites around the spinous process of the T4–T9 segment of the back. Moreover, increased PU had a positive correlation with decreased pain thresholds. One animal experimental study (Ding and Jiang, 2018) found that acupoint sensitization on the body surface was positively associated with increased PU at local sites in rats with knee osteoarthritis. Another trial (Xu et al., 2005) found that the temperature of specific sites on the body surface [i.e., acupoints in the lung meridian and Feishu (BL13) on the back] was significantly increased in patients with lung diseases. In particular, high-temperature lines distributed in the lung meridian in diverse lengths could be observed in the upper limbs, and their lengths were positively correlated with the severity of lung diseases (Xu et al., 2005).

Nevertheless, it should be pointed out that in the disease state of the lung, although the temperature of some specific sites on the body surface [i.e., Taiyuan (LU9)/Chize (LU5) of the lung meridian] in COPD patients was significantly increased compared with healthy individuals, while the overall mean temperature of the circulation line of the lung meridian in the forearm was not statistically different compared with healthy controls, indicating that the temperature for some non-acupoints of the lung meridian in the forearm was decreased. Therefore, the rules in terms of temperature changes in both acupoints and non-meridian points distributed in the circulation region of the lung meridian during the disease state of COPD need to be further investigated.

In terms of the metabolic characteristics, we found that rSO_2_ of specific sites on the body surface [i.e., Taiyuan (LU9)/Chize (LU5) in the lung meridian and Shaohai (HT3) in the heart meridian] was significantly decreased compared with healthy controls, whereas rSO_2_ of Shenmen (HT7) in the heart meridian did not change significantly, suggesting that there is relative specificity for the body surface-viscera correlation in terms of metabolic characteristics.

In addition, it should be noted that, for COPD patients in our study, the outcomes of microcirculatory characteristic (i.e., PU) and thermal characteristic (i.e., temperature) in specific sites [i.e., Taiyuan (LU9)/Chize (LU5) in the lung meridian] in the superficial layer of the skin increased significantly, whereas the metabolic characteristic (i.e., rSO_2_) in specific sites [i.e., Taiyuan (LU9)/Chize (LU5) in the lung meridian and Shaohai (HT3) in the heart meridian] in the deep layer (2 cm beneath the skin) of the body decreased significantly. The formal phenomenon can be explained by previous findings that acupoint sensitization is often accompanied by increased microcirculatory blood perfusion and temperature in the superficial layer of the body at the acupoints (Ding and Jiang, 2018), while reasons for the latter phenomenon is probably because COPD is a typical chronic disease and patients’ long-term ischemic and hypoxic states lead to a significant decrease in rSO_2_ in the deep layer of the body (Wilson et al., 2013; Buekers et al., 2019).

Last but not the least, different anatomical structures of the acupoints may be related to the above-mentioned results. The anatomical structures of Taiyuan (LU9) and Chize (LU5) of the lung meridian involve more large arteries [e.g., the radial artery under Taiyuan (LU9) and the brachial artery near Chize (LU5)], whereas the anatomical basis underlying Shenmen (HT7) and Shaohai (HT3) of the heart meridian involve fewer large arteries, such difference in anatomical structures of acupoints may have certain effects on the results discussed above. Therefore, it is likely that the anatomical basis of acupoints has certain effects on the specificity of body surface-viscera correlation in the pathological state (Rong and Zhu, 2005; Rong et al., 2011), which is also a research direction for our further fundamental research.

### 4.2 Strengths of our study

First, despite that a variety of previous studies attempted to explore the meridian-viscera correlation, the majority of them focused on the association between one meridian and one viscus (Zhang et al., 2018). There is a great lack of studies focusing on the specificity of the meridian-viscera correlation among multiple meridians and multiple viscera. Therefore, the finding of our study will shed further light on the rules of both the specificity for meridian-viscera correlation in TCM and the body surface-viscera correlation in Western medicine, thereby promoting their diagnostic and therapeutic values.

Second, in terms of measurement sites, the majority of previous studies in this field examined the biological characteristics of sites in the reaction regions of viscera (e.g., regions distributed on the back) or local sites of the impaired viscus, while the measurement sites of interest in our study focused on sites on the body surface distal from viscera. And our findings are in favor of the specificity for body surface-viscera correlation during physiological states, which will shed light on modern studies such as the emerging SPARC program (Wang et al., 2019; Ma et al., 2020).

Third, this is the first study to adopt LDF, IRT, and fNIRS at the same time to investigate diverse kinds of biological characteristics of sites on the body surface. Multiple studies have proved the accuracy and reliability of these three modern techniques and they were also widely used in this field (Wang et al., 2017; Ghafoor et al., 2019; Álvarez-Prats et al., 2019) due to advantages of non-invasiveness, rapid response, convenience, tissue specificity and reproducibility.

### 4.3 The selection basis for meridians and acupoints of interest

Due to several reasons, we selected the heart and lung meridians as targets for comparison. First, compared with other meridians, the circulation routes of these two meridians are shorter. Second, given that the circulation routes of these two meridians are adjacent in the upper limb, there are many acupoints on the same cross-section that can be compared laterally. Third, the lung is close to the heart anatomically, so these two viscera have close mutual physiopathological associations. Theoretically, acupoints of both the heart meridian and lung meridians can be used for treating heart diseases as well as lung diseases (Liu et al., 2019; Yu et al., 2020). Nonetheless, when treating lung diseases, is the therapeutic effect of stimulating the lung acupoints superior to that of stimulating acupoints of the heart meridian? In turn, when treating heart diseases, is the effect of stimulating the heart acupoints superior to that of stimulating acupoints of the lung meridian? That is to say, in terms of the therapeutic effect of acupuncture therapy, further investigations are urgently needed to investigate whether there is specificity for the modulating effect of acupoints. Taken together, it is of high priority to choose the heart meridian and lung meridian for exploring the specificity of body surface-viscera correlation.

Regarding the reasons for the selection of these four acupoints of interest, Taiyuan (LU9) and Shenmen (HT7) are located on the same cross-section of the forearm, as well as Chize (LU5) and Shaohai (HT3), thereby making them laterally comparable. Moreover, according to classic meridian theories, Taiyuan (LU9) and Shenmen (HT7) are classified as Yuan-Source acupoints, while Chize (LU5) and Shaohai (HT3) are He-Sea acupoints. Both classic meridian theories (Huang and Cheng, 2001; Yan et al., 2021) and modern research (Du et al., 2016; Peng et al., 2020) indicate that Yuan-Source acupoints and He-Sea acupoints are sensitive to manifest the pathological changes of viscera (Du et al., 2016).

### 4.4 The selection basis for COPD

After determining the selection of the lung meridian, the selection of viscera diseases directly related to the lung meridian is COPD because it is a typical kind of chronic lung disease. Previous studies have also shown that the pathological state of COPD has a significant impact on peripheral microcirculation, energy metabolism and oxygen saturation in patients (Wilson et al., 2013; Buekers and Theunis, 2019; Macrea et al., 2019), so it is more advantageous to observe corresponding changes in the biological characteristics on the body surface. In addition, the diagnostic criteria of COPD are clear and widely accepted; indicators for efficacy evaluation are standardized, which is contributive to the strict inclusion of patients and the objective detection of related indicators.

### 4.5 Limitations

First, the sample size is relatively small. Nonetheless, the results of this trial can offer a reference for the feasibility of the trial methodology and also yield preliminary data to shed light on future studies. Second, although various measures have been taken to control the influence of confounding factors on the biological characteristics of the body surface, such as environmental factors, physiological activity factors, and drug factors, it might be unable to control some individual differences and other potentially unknown factors that may affect current results. Thus, a certain caution must be taken in interpreting these results. Third, limited to the current level of technological development, for instance, the maximum number of probes that the device of LDF and fNIRS can be configured is 4, so only four sites on the body surface were detected simultaneously. The biological characteristics of sites on non-meridians or non-acupoints located in meridians were not measured. In light of more advanced techniques, more sites should be measured in future studies to investigate the specificity of the body surface-viscera correlation in a more systematic way.

## 5 Conclusion

In the disease state of COPD, the microcirculatory, thermal, and metabolic characteristics of specific sites on the body surface in the lung meridian generally manifest more significant changes than those in the heart meridian, thereby supporting the relative specificity of the body surface-viscera correlation in the pathological state.

## Data Availability

The raw data supporting the conclusion of this article will be made available by the authors, without undue reservation.
